# LncRNA MALAT1 functions as a competing endogenous RNA to regulate ZEB2 expression by sponging miR-200s in clear cell kidney carcinoma

**DOI:** 10.18632/oncotarget.5357

**Published:** 2015-10-09

**Authors:** Haibing Xiao, Kun Tang, Peijun Liu, Ke Chen, Junhui Hu, Jin Zeng, Wei Xiao, Gan Yu, Weiming Yao, Hui Zhou, Heng Li, Yingtian Pan, Anping Li, Zhangqun Ye, Ji Wang, Hua Xu, Qihong Huang

**Affiliations:** ^1^ Department of Urology, Tongji Hospital, Tongji Medical College, Huazhong University of Science and Technology, Wuhan 430030, China; ^2^ Institute of Urology, Tongji Hospital, Tongji Medical College, Huazhong University of Science and Technology, Wuhan 430030, China; ^3^ Translational Medicine Center, Tongji Hospital, Tongji Medical College, Huazhong University of Science and Technology, Wuhan 430030, China; ^4^ Department of Biomedical Engineering, Stony Brook University, Stony Brook, New York 11794, USA; ^5^ The Wistar Institute, Philadelphia, Pennsylvania 19104, USA; ^6^ Department of Cell Death and Cancer Genetics, The Hormel Institute, University of Minnesota, Austin, Minnesota 55912, USA

**Keywords:** lncRNA, MALAT1, miR-200s, ZEB2, KIRC

## Abstract

Long non-coding RNA (lncRNAs) play a critical role in the development of cancers. LncRNA metastasis-associated lung adenocarcinoma transcript 1(MALAT1) has recently been identified to be involved in tumorigenesis of several cancers such as lung cancer, bladder cancer and so on. Here, we found that MALAT1 exist a higher fold change (Tumor/Normal) in clear cell kidney carcinoma (KIRC) from The Cancer Genome Atlas (TCGA) Data Portal and a negative correlation with miR-200s family. We further demonstrated MALAT1 promote KIRC proliferation and metastasis through sponging miR-200s *in vitro* and *in vivo*. In addition, miR-200c can partly reverse the MALAT1′s stimulation on proliferation and metastasis in KIRC. In summary we unveil a branch of the MALAT1/miR-200s/ZEB2 pathway that regulates the progression of KIRC. The inhibition of MALAT1 expression may be a promising strategy for KIRC therapy.

## INTRODUCTION

Renal cell carcinoma (RCC) accounting for nearly 5% of adult malignancies with about 63,920 new cases and 13,860 deaths estimated for 2014 in the United States [1]. Clear cell kidney carcinoma (KIRC) is the most common subtype of RCC and accounts for approximately 75–80% of these tumors with the highest rates of local invasion, metastasis, mortality and refractory to current treatments [2]. Also, renal cancer patients respond poorly to radiation treatment and conventional chemotherapy [3]. Hence, a better understanding of the mechanisms involved in the pathogenesis of KIRC and more effective therapeutic approaches are instantly required.

Long noncoding RNAs (lncRNAs) are a class of transcripts longer than 200 nucleotides with limited protein coding ability [4]. Recently, many studies have shown that lncRNAs are frequently dysregulation in various tumors and have multiple functions in a wide range of biological processes, such as the proliferation, apoptosis, cell cycle arrest or cell migration and invasion [5]. LncRNA metastasis-associated lung adenocarcinoma transcript 1 (MALAT1) located on chromosome 11q13 has recently been identified to be involved in tumorigenesis of several cancers such as lung cancer, pancreatic cancer, and cervical cancer [6–12]. Hiroshi Hirata *et al* also have found that long noncoding RNA MALAT1 promotes aggressive renal cell carcinoma through Ezh2 and interacts with miR-205 [7]. However, how MALAT1 function in KIRC pathogenesis remains largely unknown.

In this study, we have found MALAT1 exist highest fold change (Tumor/Normal) in KIRC among Pan-Cancer Networks by mining clinical and expression profiles of 14 cancer types (>6000 samples) from TCGA Data Portal [13]. Our data also confirmed that MALAT1 have higher expression in renal cancer cell lines and renal cancer tissues. Additionally, our data indicated that knockdown expression of MALAT1 decreased renal cancer cell proliferation, migration and invasion *in vivo* and *in vitro*. MALAT1 functions as an oncogene through sponging miR-200s and increasing the expression of ZEB2. Together, these data contribute to the characterization of the molecular mechanisms of KIRC progression.

## RESULTS

### MALAT1 was up regulated in KIRC

**Figure 1:**
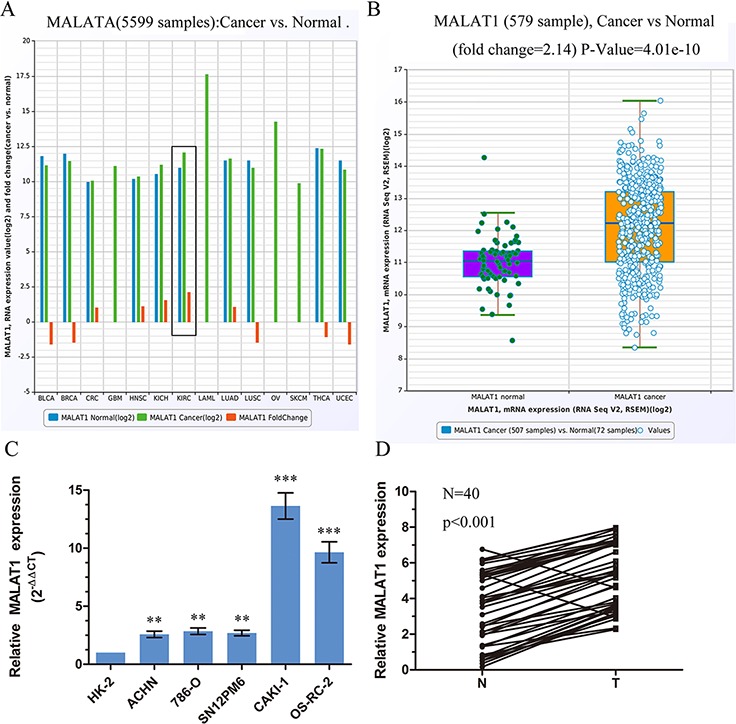
MALAT1 was upregulated in ccRCC **A.** The expression of MALAT1 among Pan-Cancer including 14 cancer types from The Cancer Genome Atlas (TCGA) Data Portal from starBASE v2.0 (http://starbase.sysu.edu.cn/panCancer.php). The black box means the expression of MALAT1 in normal or clear cell kidney carcinoma. **B.** The expression of MALAT1 in normal or clear cell kidney carcinoma from TCGA Data Portal. **C.** Real-time PCR analysis of MALAT1 expression in immortalized human renal tubule epithelial cell line HK-2 and indicated renal carcinoma cell lines. **D.** Relative expression of MALAT1 in 40 pairs of ccRCC tumor tissues and their corresponding adjacent non-cancerous tissues. The average MALAT1 expression was normalized by GAPDH expression. **P < 0.01; ***P < 0.001.

To determine whether MALAT1 was involved in the tumorigenesis or development of KIRC, we firstly examined the expression of MALAT1 in TCGA Data Portal from starBASE v2.0 (http://starbase.sysu.edu.cn/panCancer.php) and found MALAT1 exist higher expression in tumor than in normal in Chromophobe renal cell carcinoma (KICH) and clear cell kidney carcinoma (KIRC) (Figure 1A). MALAT1 expression was significantly upregulated in cancer tissues (mean ratio of 2.14-fold, *P* < 0.01) compared with normal counterparts in KIRC (Figure 1B). To further support this conclusion, we examined the expression of MALAT1 in renal cancer cell lines, renal cancer tissues and their corresponding noncancerous tissues from Tongji Hosptial. Real-time PCR analysis demonstrated that MALAT1 was ubiquitously expressed at higher levels in a panel of 5 human clear cell renal cell carcinoma lines than immortalized human proximal renal tubule epithelial cell line HK-2 (Figure 1C). In parallel, as showed in Figure 1D, qRT-PCR showed that MALAT1 was significantly upregulated in renal cancer tissues (*p* < 0.001). Patient characteristics are in Table S1. Taken together, these results suggested that MALAT1 may play an important role in KIRC progression.

### Knockdown of MALAT1 inhibited cell proliferation and metastasis *in vitro*

**Figure 2:**
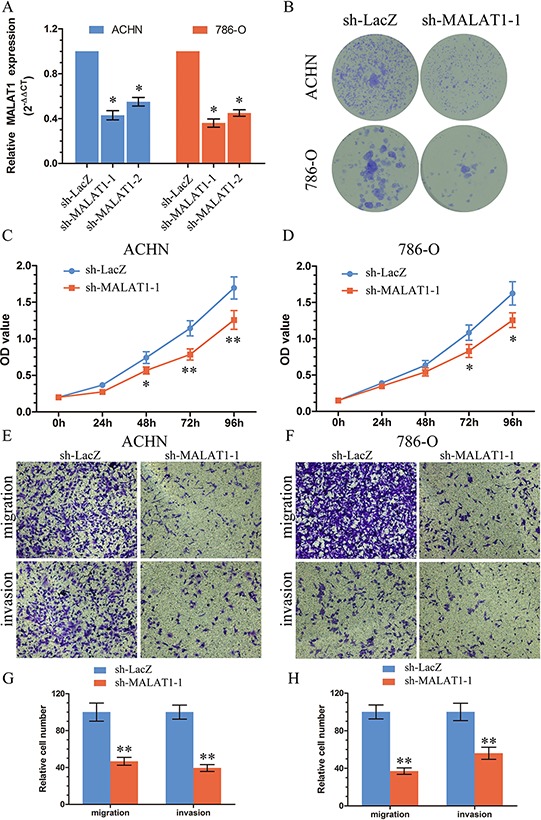
Knockdown of MALAT1 inhibited cell proliferation and metastasis *in vitro* **A.** The efficiency of MALAT1 silencing in short hairpin RNA-stably transduced renal cancer cell lines ACHN and 786-O. Relative gene expression was determined using the comparative delta-delta CT method (2^−ΔΔCt^). **B.** Representative micrographs of crystal violet-stained cell colonies analyzed by clongenic formation. **C–D.** MTS assays revealed cell growth curves of indicated cells. **E–F.** Migration and invasion assay for renal cancer cells. Representative photographs were taken at × 200 magnification; number of migrated cells was quantified in ten random images from each treatment group. **G–H.** Results were the mean ± SEM from two independent experiments and plotted as percent (%) cells relative to sh-LacZ or sh-MALAT1–1. *P < 0.05; **P < 0.01.

To explore the role of MALAT1 in renal cancer cells, we stably inhibited MALAT1 in two KIRC cell lines ACHN and 786-O with lenti-viruses carrying shRNA for MALAT1 and a control nonspecific shRNA (LacZ) (Figure 2A). Colony formation assay (Figure 2B) and MTS (Figure 2C, 2D) assay showed that knockdown MALAT1 inhibited cell proliferation in ACHN and 786-O cells. Further assay of transwell showed that knockdown MALAT1 suppressed renal cancer cell migration and invasion (Figure 2E, 2F, 2G and 2H). Western Blot showed that MALAT1 can influence the expression of E-cadherin, N-cadherin and Vimentin (Figure S1).

### miR-200s bind to and suppress MALAT1 expression

**Figure 3:**
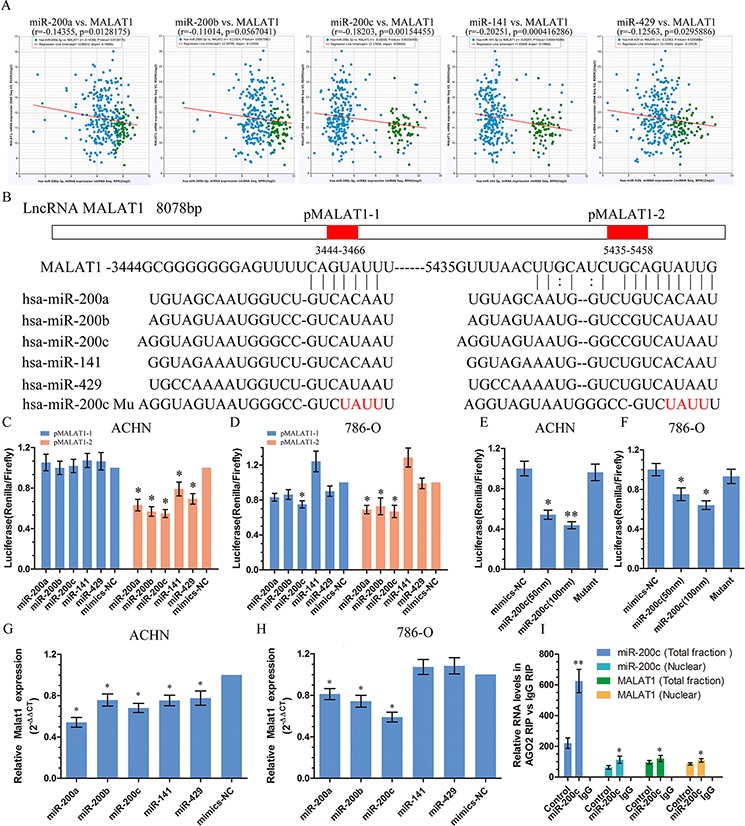
MiR-200s bound to and suppressed MALAT1 expression **A.** MALAT1 expression correlates with miR-200s family expression in clear cell kidney carcinoma (KIRC) from TCGA Data Portal from starBASE v2.0 (http://starbase.sysu.edu.cn/panCancer.php). **B.** Schematic miR-200s putative target sites in 3′ UTRs of MALAT1. The red part in the column implies the possibly binding sites in MALAT1 were named pMALAT1–1 and pMALAT1–2. The red word means the mutant area of miR-200c. **C, D, E, F.** Luciferase reporters harboring putative target sites in the 3′ UTRs of pMALAT1–1 and pMALAT1–2 were co-transfected with 50 and 100 nM of indicated small RNA molecules in ACHN and 786-O cells. Relative luciferase activity was plotted as the mean ± SEM of three independent experiments. **G, H.** The relative expression of MALAT1 in renal cancer cell ACHN and 786-O after transfected with miR-200s family. **I.** The amount of miR-200c and MALAT1 bound to AGO2 was measured by qPCR in nuclear and total fractions in presence of a negative control for mimics or miR-200c mimics.

Recently, many RNA transcripts have been reported to function as competing endogenous RNAs (ceRNA) by competitively binding common microRNAs [5, 14, 15]. Growing evidence supports an important role for miR-200s family in KIRC [16, 17]. It existed antagonistic effect of MALAT1 and miR-200s family on cell proliferation and metastasis in KIRC. Then we found that MALAT1 expression correlates with miR- 200s family expression in human renal cancer tissue from TCGA Data Portal (Figure 3A). We used starBase v2.0 to predict the interaction between miR-200s and MALAT1 and it showed that there were two binding sites in MALAT1. We cloned them to psiCkeck2 vector and named pMALAT1-1 and pMALAT1-2 (Figure 3B). The efficiency of transfection is examined by real-time PCR in Figure S2. After co-transfection with pMALAT1-1 or pMALAT1-2 and miR-200s family, there were an obvious decrease in luciferase activity compared with the negative control in pMALAT1-2 in ACHN (Figure 3C). There were also lower activities of luciferase reporter in co-transfected with pMALAT1-2 and miR-200a, miR-200b or miR-200c in 786-O compared with the negative control (Figure 3D). We also found that co-transfected with miR-200c and pMALAT1-2 decrease luciferase activity in 786-O (Figure 3D). Since miR-200c had obvious effects on MALAT1, we further designed miR-200c mutant (Figure 3B) and co-transfected with pMALAT1-2 to ACHN and 786-O. However, there were no effects on the luciferase reporter activities of MALAT1. At the same time, reporter assays showed that the activity of luciferase linked with the pMALAT1-2 was repressed in a dose-dependent manner in miR-200c mimics–transfected ACHN and 786-O cells, compared with those in control cells (Figure 3E, 3F). To further support this conclusion, we transfected miR-200s family and then detected the expression of MALAT1 in ACHN and 768-O. The expression of MALAT1 was widely decreased in ACHN after transfected with miR-200s family and 786-O after transfected with miR-200a, miR-200b and miR-200c (Figure 3G, 3H), whereas down-regulation of MALAT1 can up-regulate the expression of miR-200c (Figure S3), suggesting a direct interaction between MALAT1 and miR-200s. Given that MALAT1 is localized in nuclear and miRNAs mainly exert their functions in cytoplasm, we performed *in situ* hybridization to confirm the existence of miR-200c in the nucleus. As shown in Figure S4, miR-200c was found to be localized in both in the cytoplasm and the nucleus of ACHN cells. It is believed that miRNAs are able to regulate coding and non-coding RNA by recruiting nuclear localized AGO2 [11, 18]. Therefore, we further employed an AGO2 RIP assay and found that miR-200c mimics transfection caused a enrichment of MALAT1 in AGO2 immunoprecipitates in both total and nuclear extracts (Figure 3I). Taken together, these results indicate that nuclear localized miR-200s could directly bind to MALAT1 and suppress the expression of MALAT1 through an Ago2- dependent manner.

### MALAT1 upregulates ZEB2 levels

**Figure 4:**
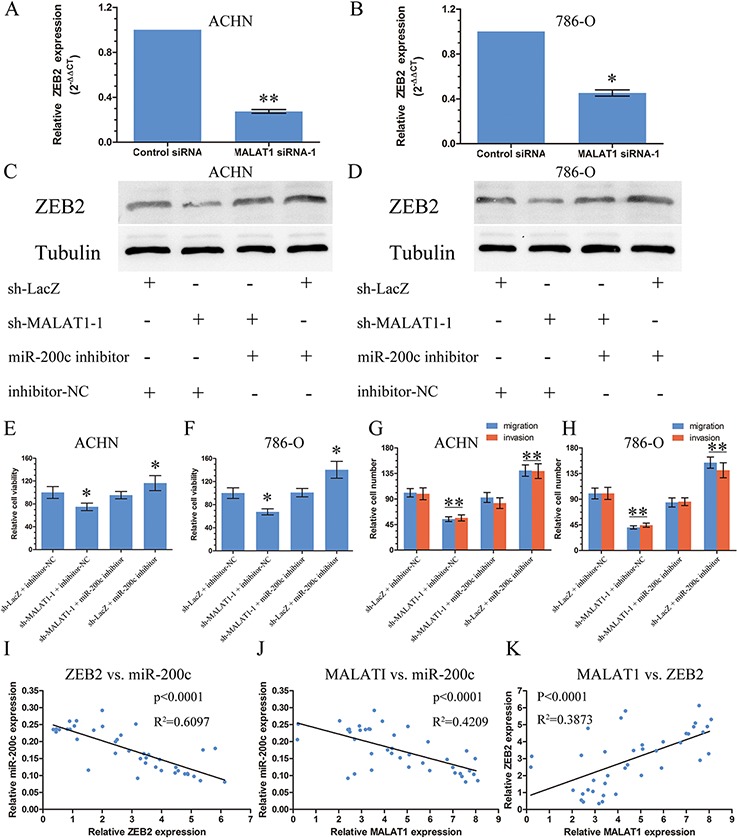
MALAT1 upregulated ZEB2 Level **A** and **B.** The expression of ZEB2 in renal cancer cell lines ACHN and 786-O after transfection with sh-MALAT1 or sh-LacZ by real-time PCR. **C** and **D.** The expression of ZEB2 in renal cancer cell lines ACHN and 786-O after co-transfected with sh-MALATI-1/sh-LacZ or miR-200c inhibitor/negative control for inhibitor by western blot. **E** and **F.** The proliferation assays were performed to evaluate the effect of MALAT1 on the function of miR-200c. **G** and **H.** The migration and invasion assays were performed to evaluate the effect of MALAT1 on the function of miR-200c. **I, J** and **K.** The correlation of MALAT1, ZEB2 and miR-200c in renal cancer tissue from Tongji Hosptial. *, *P* <0.05; **, *P*<0.01.

Among the many targets of miR-200s family, we concentrated on ZEB2 since it is a member of the Zfh1 family of 2-handed zinc finger/homeodomain proteins with a significant function in metastasis among KIRC [19]. It had six domains that can be bound by miR-200c predicted by TargetScan and had a strongly negative correlation with miR-200c from TCGA Data Portal (Figure S5). The expression of ZEB2 was inhibited after transfected with sh-MALAT1 compared with the control by real-time PCR in ACHN and 786-O (Figure 4A and 4B). Furthermore, the effects of MALAT1 expression on endogenous ZEB2 protein were monitored. It showed that sh-MALAT1 can inhibit the expression of ZEB2 whereas mir-200c inhibitor can relieve the inhibition of ZEB2 by MALAT1 (Figure 4C and 4D). To further establish a functional connection between miR-200c and MALAT1, we tested whether MALAT1 deregulation was required for regulation of miR-200c on cell proliferation and metastasis. We transfected miR-200c inhibitor or negative control of the inhibitor to renal cancer cells stably transfected with sh-MALAT1–1 or sh-LacZ. We found that the effect of miR-200c inhibitor was partially attenuated by sh-MALAT1–1 on proliferation, migration and invasion (Figure 4E, 4F, 4G and 4H).

We then detected the expression of ZEB2, miR-200c and MALAT1 in 40 tumor specimens and their paired normal adjacent tissues by real-time PCR. Bivariate correlation analysis showed that expression of ZEB2 and MALAT1 was significantly correlated with miR-200c transcript level of KIRC tissues (Figure 4I, 4J). There was a positive correlation between ZEB2 and MALAT1 (Figure 4K).

Together these data indicated that by binding miR-200s family, especially miR-200c, MALAT1 acts as a ceRNA for the target ZEB2 mRNA thereby modulating the derepression of ZEB2 and imposing an additional level of post-transcriptional regulation.

### MALAT1 induces proliferation and metastasis *in vivo*

**Figure 5:**
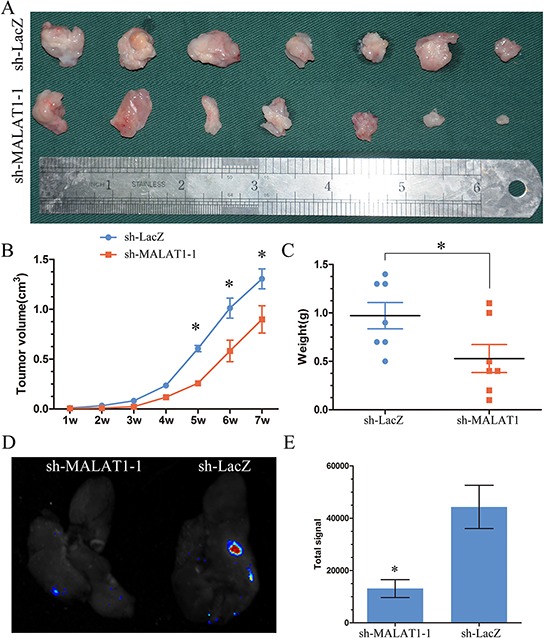
MALAT1 induced proliferation and metastsis *in vivo* **A.** Photographs of tumors excised 42 days after inoculation of stably transfected cells ACHN into nude mice. **B.** Mean tumor volume measured by caliper on the indicated days. **C.** Tumor weight of each nude mouse at the end of 42 days. **D.** Representative bioluminescent images of lungs of nude mice at the 30th day after intravenous injection of renal cancer cell ACHN. **E.** Quantification analysis of fluorescence signal from captured bioluminescence images. *P < 0.05.

MALAT1's function has never been studied in KIRC *in vivo*. To study the effect of MALAT1 on the proliferation, ACHN cells transfected with the sh-MALAT1 or sh-LacZ were used in a nude mice xenograft model. Up to 42 days, there was a dramatic decrease in tumor volume and weight in the sh-MALAT1 group compared with sh-LacZ group (Figure 5A, 5B and 5C).

As for MALAT1 played an important role in migration and invasion in KIRC *in vitro*, to further determine whether MALAT1 could promote metastatic behaviors *in vivo*, ACHN stably expressing sh-MALAT1 were delivered into nude mice by tail vein injection. We found that the total signal of metastatic nodules in the lung was dramatically decreased in Lenti-sh-MALAT1 groups when compared with sh-Lacz group (Figure 5D and 5E).

These results suggest that the level of MALAT1 expression is significantly associated with the proliferation and metastasis capacity of KIRC *in vivo*.

## DISCUSSION

A majority of lncRNA that are expressed at low levels and in general poorly conserved and nearly one-third of them are primate specific [20, 21]. However, unlike these lncRNA, MALAT1 is extremely abundant, ubiquitously expressed and highly conserved among mammals, reaching up to 90% identity between human and mouse. In addition, this transcript seems to be very stable due to a triple helix in the 3′end, which prevents its degradation [22, 23]. MALAT1 mainly focused on regulates alternative splicing by modulating the levels of active serine/arginine proteins [24]. Additionally, MALAT1 can bind to unmethylated Polycomb 2 protein promotes E2F1 SUMOylation, leading to activation of the growth-control gene program [25]. MALAT1 can also active chromatin sites [26]. Recently, it reported that MALAT1 can act as an oncogene through Ezh2 and interacts with miR-205 in KIRC [6, 7]. Nevertheless, tumorigenic properties and mechanistic heterogeneity of MALAT1 are far from being fully elucidated in KIRC. Competing endogenous RNAs (ceRNAs) are endogenous transcripts that, irrespectively of their ability to encode for a protein, share common microRNA Recognition Elements (MREs) and hence compete for the binding of usual miRNA molecules. The outcome of such competition is that ceRNAs relieve each other from microRNA-mediated inhibition and positively impact each other's expression levels [15]. And we were preparing this manuscript pseudogene PTENP1 functions as a competing endogenous RNA to suppress clear cell renal cell carcinoma progression [5]. Inspired by the ceRNA regulatory network and emerging evidence that suggests that MALAT1 may participate in this regulatory circuitry as for its extremely abundant, ubiquitously expressed [9, 11, 27], we hypothesized that MALAT1 may also serve as a ceRNA and so we searched for potential interactions with miRNAs.

**Figure 6:**
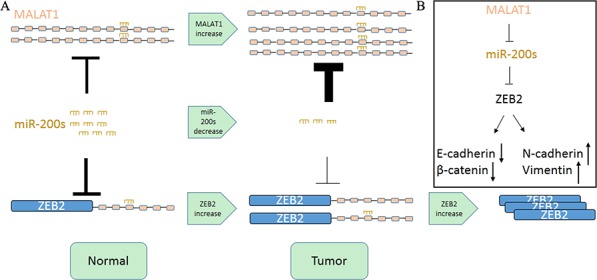
Schematic Model of MALAT1 Functions as a ceRNA to regulate ZEB2 expression by sponging miR-200s in KIRC **A.** MALAT1 promotes KIRC cell proliferation and metastasis by competitively binding the miR-200 family, upregulating ZEB2. **B.** MALAT1 also regulates the expression of four other target genes via ceRNA crosstalk.

In this study, we first examined the expression of MALAT1 in TCGA Data Portal from starBASE v2.0 (http://starbase.sysu.edu.cn/panCancer.php) and found MALAT1 exist highest fold change (Tumor/Normal) in KIRC among the Pan-cancer network. We then further verified this in renal cancer cell lines and 40 paired renal cancer tissue and their adjacent tissue from Tongji Hosptial. We then found that knockdown MALAT1 can inhibit renal cancer cell proliferation and metastasis *in vitro* and *in vivo*. As for miR-200s existed a lower expression and played an important role in proliferation and metastasis in carcinogenesis and it showed a widely negative correlation among miR-200s family and MALAT1 except miR-200b with a P value 0.056 in TCGA Data Portal. We employed bioinformatics analysis and founded that MALAT1 may exist two binding sites with the miR-200s family. We then cloned the two binding sites to the psiCheck2 vector separately and co-transfected with miR-200s family. In renal cell line ACHN, the miR-200s family can widely combine with the second binding site. However, in renal cancer 786-O, miR-200c can bind to the binding site both, whereas the miR-200a and miR-200b can only bind to the second binding site. Maybe the second binding site had longer seed sequence and lower free energy. To further investigate the luciferase activity, we chose miR-200c as a model miRNA for further studies and found miR-200c can bind to MALAT1 in a dose dependent whereas miR-200c mutant can't bind to the MALAT1. We also found that miR-200s family can reduce the expression of MALAT1 by real time PCR in ACHN. While in 786-O, miR-200b and miR-200c can inhibit the expression of MALAT1. Maybe the different background of renal cancer exists different results. ZEB2 is a member of the Zfh1 family of 2-handed zinc finger/homeodomain proteins located in the nucleus and functions as a DNA-binding transcriptional repressor that interacts with activated SMADs and widely reported target of miR-200c [28]. We found that knockdown MALAT1 can inhibit the expression of ZEB2 by real time PCR. We also found that knockdown MALAT1 can decrease the expression of ZEB2 whereas miR-200c inhibitor can partly reverse the reduction cause by knockdown MALAT1. What's more, we demonstrated that miR-200c, MALAT1 and ZEB2 existed obviously correlation in KIRC. Recently Paola Paci *et. al* reported that PVT1 could function as a ceRNA in normal breast tissues by sponging miR-200s [29]. These authors also demonstrated that some ceRNA interactions appear to be turned-on in normal breast tissues (e.g. PVT1-miR-200s) and some ceRNA interactions appear to be turned-off in cancer breast tissues, which suggested a marked rewiring in the ceRNA program between normal and pathological breast tissue [29]. Consistent with this study, we also found that MALAT1 can also influence the expression of ZEB2 through binding with miR-200c in normal renal cell line HK-2 (Figure S6). In fact, it is reported that ZEB2 overexpression is an independent biomarker for the poor prognosis of patients with RCC [30]. The promoter of ZEB2 can be bound by Forkhead box Q1, FOXA2, AP-1 and so on [31–33] and the expression of mRNA can be regulated. However as showed in Figure 6, the expression of ZEB2 can be further inhibited by miR-200s as for its differential expression in KIRC. In KIRC, miR-200s exist generally lower expression in tumor tissue comparing with para-carcinoma tissue [34]. What's more, the high abundance and stability of MALAT1 and its differential expression can aggravate the expression of ZEB2 in KIRC through miR-200s.

In conclusion, our results have provided strong evidence that MALAT1 can function as a competing endogenous RNA to promote ZEB2 expression by sponging miR-200s and potentially serve as a therapeutic target in KIRC.

## MATERIALS AND METHODS

### Human samples

A total of 40 paired clear cell renal cell carcinoma and corresponding noncancerous tissues were obtained sequentially from patients undergoing radical nephrectomy from the period of 2010–2014 in Tongji hospital. Corresponding noncancerous tissues were acquired at least 5 cm away from the tumor site.

### Cell culture, infection and transfection

ACHN, 786-O, SN12-PM6 and HK-2 cells were maintained in Dulbecco's modified Eagle's medium supplemented with 10% fetal bovine serum and 2 mmol/l l-glutamine in a humidified atmosphere of 5% CO2 maintained at 37°C. OS-RC-2 and CaKi-1 cells were cultured in RPMI-1640 supplemented with 10% fetal bovine serum and 2 mmol/l l-glutamine. Oligonucleotides (miRNA mimics, negative control of mimics, miRNA inhibitor and negative control of inhibitor) were ordered from RiboBio (China). Cells were seeded into plate wells and incubated overnight, and then 50 nM of small RNA molecules were transported into cells by using X-tremeGENE (Roche). Two putative MALAT1 target sites were cloned into the XhoI-NotI site of the dual luciferase Psicheck2 plasmid (Promega) separately. Oligos corresponding to the target sequences were annealed and cloned into the HpaI and XhoI sites of the pSicoR plasmid (Addgene). The following target regions were chosen: MALAT1–1#, GGGAGTTACTTGCCAACTTG; MALAT1–2#, CCAGGCTGGTTATGACTCAG.

### Quantitative real-time PCR (qRT-PCR) and western blot analysis

Target genes and controls were analyzed by qRT-PCR using SYBR Premix Ex TaqTM (TaKaRa, Dalian, China). The details of western blot and antibodies were under supplementary materials and methods.

### Cell viability, migratory and invasion assays

Cell viability was performed as previously described [35]. It was assessed at 0, 24, 48, 72 and 96 hours upon treatments by the 3-(4,5-dimethylthiazol-2-yl)-5-(3-carboxymethoxyphenyl)-2-(4-sulfophenyl)-2H-tetrazolium, inner salt (MTS) method (Sigma, USA) according to the manufacturer's instructions. The MTS have six replications. The colony formation has three replications. The details of the protocol of colony formation and migratory and invasion assays were under Supplementary materials and methods.

### Xenograft subcutaneously and experimental lung metastasis model

Two groups of eight mice each were injected subcutaneously with prepared cells at the same site. Tumor volume was calculated using the formula, V = 0.5ab^2^, where a represents the larger and b represents the smaller of the two perpendicular indexes. Animals were killed 42 days after injection and tumors were weighed. The details of experimental lung metastasis model were under supplementary materials and methods.

### Luciferase assays

Briefly, ACHN and 786-O cells were seeded in 48- well plates (5000 cells per well) and co-transfected with 100 ng psicheck2 Luciferase vectors containing target genes 3′UTR with 50 nM miR-200s mimic or mutant mimic or NC. Forty-eight hours after transfection, Dual- Luciferase Reporter Assay (Promega) were performed according to the manufacturer's instructions, as previously described.

### RNA immunoprecipitation

RNA immunoprecipitation was performed as previously described [11, 36]. Magnetic beads conjugated with human anti-Ago2 antibody and negative control normal mouse IgG were from Millipore. Nuclei were isolated from ACHN cells 24 hours after transfection using Nuclei EZ prep (Sigma) according to the manufacturer's instructions.

### Statistical analysis

Continuous data were compared using Student's 2-tailed t test. Data are represented as mean ± SEM. In all cases, *P* < 0.05 was considered statistically significant. *p < 0.05; **p < 0.01; ***p < 0.001.

## SUPPLEMENTARY FIGURES AND TABLE


