# Supplementary material for: LncRNA MALAT1 functions as a competing endogenous RNA to regulate ZEB2 expression by sponging miR-200s in clear cell kidney carcinoma
Source: Oncotarget. 2015 Oct 9;6(35):38005–15. doi: 10.18632/oncotarget.5357 (PMC4741980; doi:10.18632/oncotarget.5357)
Supplement: Supplementary file 1 [file oncotarget-06-38005-s001.pdf]

# SUPPLEMENTARY FIGURES AND TABLE

## Quantitative real-time PCR (qRT-PCR) and Western blot analysis

Total RNA of tissues and cells was extracted with TRIzol reagent (Invitrogen, Carlsbad, CA) according to the manufacturer's protocol with modification. Reverse transcription of microRNA and mRNA were done using RevertAid™ First Strand cDNA Synthesis Kit (Fermentas, Vilnius, Lithuania) and miProfile™ miRNA qPCR Primer (GeneCopoeia, Guangzhou, China). qRT-PCR analysis was performed with the Platinum SYBR Green qPCR Supermix UDG kit (Invitrogen, Carlsbad, CA) using synthesized primers from GeneCopoeia (Guangzhou, China). The primer for pMALAT1–1 F: CCGCTCGAGGCCAAGTCCTGGAGAAATAGT, R: CGCGCGGCCGCCCCTCTCTCTTCCCTGTTAAG. The primer for pMALAT1-2 F: CCGCTCGAGTCCTGGAA TAAAAGAAGCCGA R: CGCGCGGCCGCGCACAT CATGCTATTCCTTTC. The primer of MALAT1 for real-time PCR F: AAAGCAAGGTCTCCCCACAAG R: GGTCTGTGCTAGATCAAAAGGCA. ZEB2 F: TGAGG ATGACGGTATTGC R: ATCTCGTTGTTGTGCCAG. Cells were prepared by washing with PBS. Protein extraction and immunoblot analysis was performed as previously described [1]. Primary antibodies ZEB2 (1:1000 dilution; GeneTex) and GAPDH (1:2000 dilution; Boster Wuhan China).

## Cell viability, migratory and invasion assays

Exponentially growing cells were plated at approximately 1,000 cells per well in 6-well plates and after treatment. Culture medium was changed every 3 days. Colony formation was analyzed 10 days following infection by staining cells with 0.05% crystal violet solution for 20 min. The 24-well transwell plate with 8 μm pore polycarbonate membrane inserts (Corning, New York, USA) was used to analyze the migration and invasive potential of cells according to manufacturer's protocol with three replications. For invasion assay, the membrane was coated with the matrigel (200 ng/ml) (BD Biosciences, Bedford, MA). For ACHN, the cell number is 100000 and time is 12 hours, as for 786-O, the cell number is 3*10000 and time is 8 hours. After 12 hours of incubation, cells invading into the lower surface of the membrane insert were fixed in 100% methanol, stained with 0.05% crystal violet, and quantified by counting in 10 random fields

## Experimental lung metastasis model

ACHN cell was stably infected with lenti-siMALAT1–1 or lenti-NC containing green fluorescent protein label. Treated cells ($2 \times 10^5$) were suspended in 100 μl of phosphate-buffered saline and injected intravenously via the tail vein. Mice were killed and lungs were resected 35 days later after injection. The incidence and volume of metastases were estimated by imaging of mice for bioluminescence using the Living Image software (Xenogen, Baltimore, MD). The photon emission level was used to assess the relative tumor burden in the mice lungs. All animal studies were conducted under approved guidelines of the animal care and use committee of the Tongji Hospital.

## Fluorescent *in situ* hybridization for detection of miR-200c

Fluorescent *in situ* Hybridization (FISH) for miR-200c, was conducted as previously described[2]. On 4% paraformaldehyde (PFA) fixed slides with monolayers of ACHN cell lines, using a 5′-DIG-labelled probe at 50 nM concentration. Hybridization was performed at 55°C for 1 hour, followed by visualization using anti-DIG-POD (Roche Applied Science) and FITC-labeled TSA (Perkin Elmer). Slides were subsequently counterstained using DAPI.

# REFERENCES

1. Li H, Wang J, Xiao W, Xia D, Lang B, Yu G, Guo X, Guan W, Wang Z, Hu Z, Liu J, Ye Z, Xu H. Epigenetic alterations of Kruppel-like factor 4 and its tumor suppressor function in renal cell carcinoma. Carcinogenesis. 2013; 34:2262–2270.

2. Leucci E, Patella F, Waage J, Holmstrom K, Lindow M, Porse B, Kauppinen S, Lund AH. microRNA-9 targets the long non-coding RNA MALAT1 for degradation in the nucleus. Scientific reports. 2013; 3:2535.

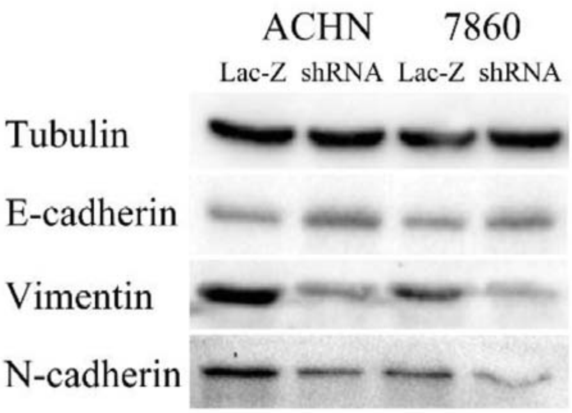


**Supplementary Figure S1: The effect of MALAT1 to metastasis relative molecules.** After transfection with sh-Lacz or sh- MALAT1–1, the expression of E-cadherin, N-cadherin and Vimentin were detected by Western blot.

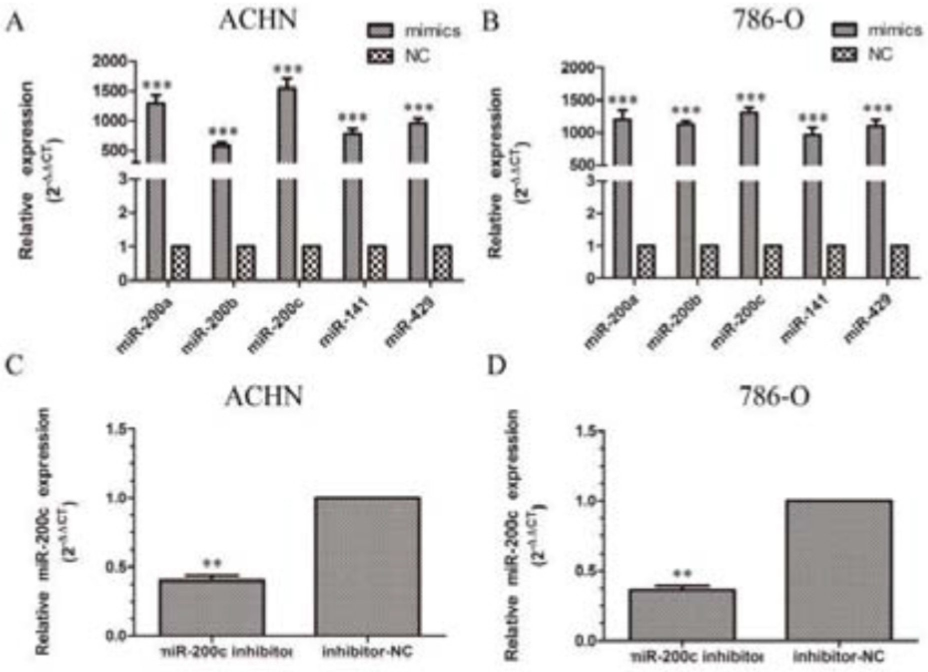


**Supplementary Figure S2: The efficiency of transfection is examined by real-time PCR.** **A, B.** After transfection with miR-200a, miR-200b, miR-200c, miR-141, miR-429 mimics or the negative control of mimics, the expression of miRNAs were detected by real-time PCR in ACHN and 786-O separately. **C, D.** The expression of miR-200c was detected by real-time PCR after transfection with miR-200c inhibitor or negative control of inhibitor. ***P* < 0.01; ****P* < 0.001.

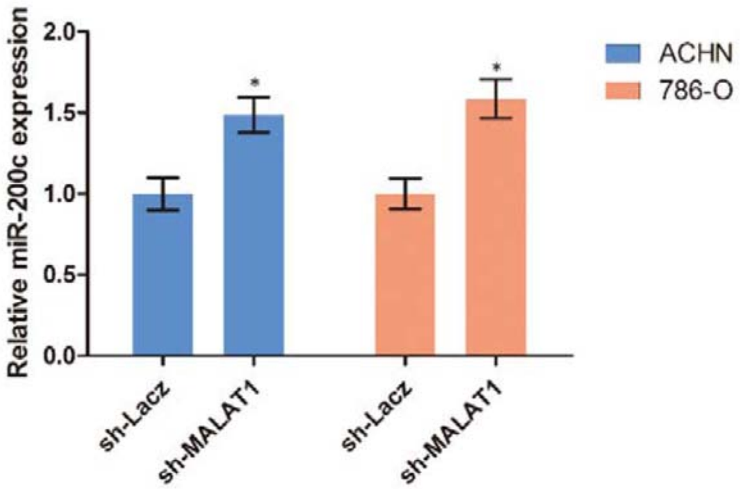


**Supplementary Figure S3: The effects of MALAT1 to miR-200c.** After transfection with sh-Lacz and sh-MALAT1-1, the expression of miR-200c were detected by RT-PCR.

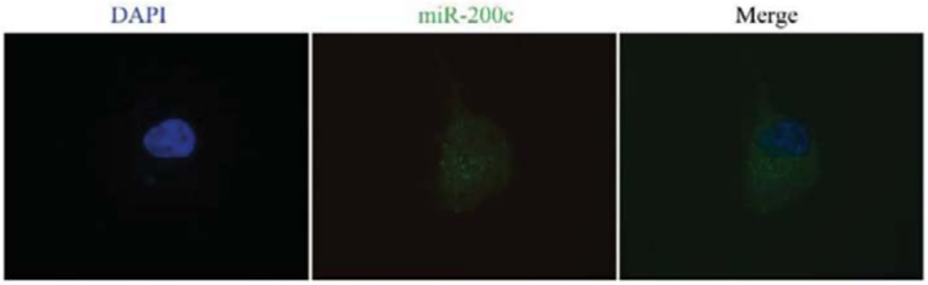


**Supplementary Figure S4: The localization of miR-200c in renal cancer cell lines.** RNA fluorescent *in situ* hybridization for miR-200c in ACHN cell lines. Magnification 200X. DAPI is in blue and miR-200c in green.

A

| | predicted consequential pairing of target region (top) and miRNA (bottom) | seed match | site-type contribution | 3' pairing contribution | local AU contribution | position contribution | TA contribution | SPS contribution | context+ score | context+ score percentile | conserved branch length | $P_{CT}$ |
|---|---|---|---|---|---|---|---|---|---|---|---|---|
| Position 392-398 of ZEB2 3' UTR<br>hsa-miR-200c | 5' ...CGCACUACCAUACAU--CAGUAUUU...<br>\|\|\| \|\|\|\|\|\|\|<br>3' AGGUAGUAAUGGGCCGUCAUAAU | 7mer-m8 | -0.120 | -0.016 | -0.111 | -0.006 | 0.013 | 0.062 | -0.18 | 91 | 1.417 | 0.35 |
| Position 455-462 of ZEB2 3' UTR<br>hsa-miR-200c | 5' ...CGCACUACAAUGCAUCAGUAUUA...<br>\|\|\|\|\|\|\|<br>3' AGGUAGUAAUGGGCCGUCAUAAU | 8mer | -0.247 | -0.008 | -0.033 | 0.001 | 0.023 | 0.096 | -0.17 | 89 | 1.549 | 0.70 |
| Position 813-820 of ZEB2 3' UTR<br>hsa-miR-200c | 5' ...UUGAAGCACCCAUGUCAGUAUUA...<br>\|\|\|\| \|\|\|\|\|\|\|<br>3' AGGUAGUAAUGGGCC--GUCAUAAU | 8mer | -0.247 | -0.018 | 0.009 | 0.091 | 0.023 | 0.096 | -0.05 | 55 | 2.010 | 0.87 |
| Position 898-905 of ZEB2 3' UTR<br>hsa-miR-200c | 5' ...GUUCAUUAAUUUUGUCAGUAUUA...<br>\|\|\|\|\|\| \|\|\|\|\|\|\|<br>3' AGGUAGUAAUGGGCC--GUCAUAAU | 8mer | -0.247 | -0.050 | -0.044 | 0.113 | 0.023 | 0.096 | -0.11 | 76 | 2.010 | 0.87 |
| Position 1029-1036 of ZEB2 3' UTR<br>hsa-miR-200c | 5' ...UCAUACUGUAGUGUACAGUAUUA...<br>\|\|\|\|\|\|\|<br>3' AGGUAGUAAUGGGCCGUCAUAAU | 8mer | -0.247 | 0.003 | -0.083 | 0.146 | 0.023 | 0.096 | -0.06 | 61 | 2.010 | 0.87 |
| Position 1363-1369 of ZEB2 3' UTR<br>hsa-miR-200c | 5' ...AAUUGUCUUUGUAAAAGUAUUAU...<br>\|\|\|\|\|\|<br>3' AGGUAGUAAUGGGCCGUCAUAAU | 7mer-1A | -0.074 | 0.001 | -0.046 | 0.099 | 0.014 | 0.069 | > -0.01 | 16 | 1.948 | 0.24 |

B

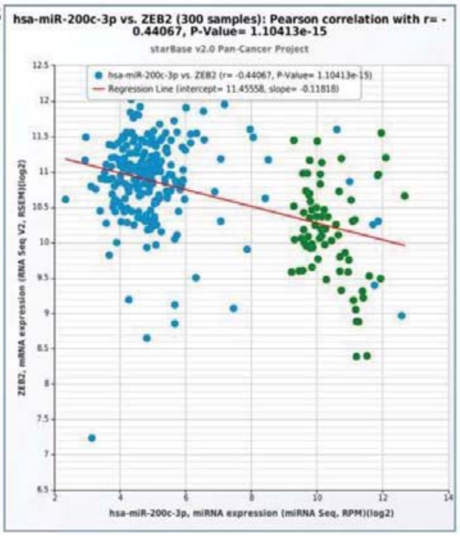


**Supplementary Figure S5: The correlation of miR-200c and MALAT1. A.** ZEB2 has six binding sites that can be combined by miR-200c predicted by TargetScan. **B.** There is a negatively correlation between miR-200c and ZEB2 from TCGA data portal.

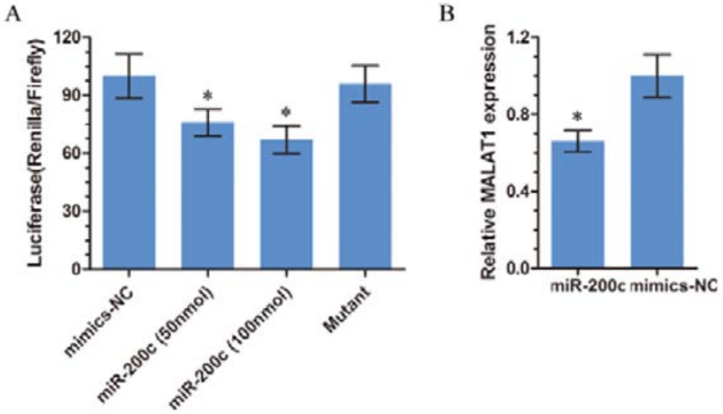


**Supplementary Figure S6: MiR-200s bound to and suppressed MALAT1 expression in HK-2 cells. A.** Luciferase reporters harboring putative target sites in the 3′ UTRs of pMALAT1-2 were co-transfected with 50 and 100 nM of indicated small RNA molecules in HK-2 cells. **B.** The relative expression of MALAT1 in HK-2 after transfected with miR-200c.

## Supplementary Table S1: Characteristics of 40 patients

| | | No of case (%) |
|---|---|---|
| Age(y) | >45 | 20(50) |
| | < =45 | 20(50) |
| Gender | Male | 22(55) |
| | Female | 18(45) |
| Clinical Stage | I | 26(65) |
| | II | 10(25) |
| | III | 3(7.5) |
| | IV | 1(2.5) |
| T classification | T1 | 25(62.5) |
| | T2 | 11(27.5) |
| | T3 | 4(10) |
